# Qualitative experiences, values, and decisional needs of patients with unprovoked venous thromboembolism who suffer bleeding—“This pill will keep you alive tonight”

**DOI:** 10.1016/j.rpth.2024.102360

**Published:** 2024-03-01

**Authors:** Maria A. de Winter, Yan Xu, Dawn Stacey, Philip S. Wells

**Affiliations:** 1 Department of Acute Internal Medicine, University Medical Center Utrecht, Utrecht, The Netherlands; 2 Department of Internal Medicine, Diakonessenhuis, Utrecht, The Netherlands; 3 Department of Medicine, University of Ottawa, The Ottawa Hospital, Ottawa, Ontario, Canada; 4 Clinical Epidemiology Program, Ottawa Hospital Research Institute, Ottawa, Ontario, Canada; 5 Faculty of Health Sciences, University of Ottawa, Ottawa, Ontario, Canada

**Keywords:** anticoagulation, hemorrhage, interviews, pulmonary embolism, venous thrombosis

## Abstract

**Background:**

Contemporary guidelines recommend extended-duration anticoagulation among patients with a first unprovoked venous thromboembolism (VTE). Little is known about whether this recommendation aligns with patient values after a bleeding complication.

**Objectives:**

To explore the experiences, values, and decisional needs of patients with unprovoked VTE related to extended-duration treatment after an anticoagulant-associated bleed.

**Methods:**

In this descriptive, qualitative study, face to face online semistructured interviews were conducted with patients with unprovoked VTE who had experienced bleeding and continued anticoagulant treatment in one academic hospital in Canada. Data were analyzed using directed content analysis to identify themes. Themes were mapped onto the Ottawa Decisional Support Framework to identify decisional needs.

**Results:**

Between September and December 2021, 14 patients were interviewed (age 41-69 years; 9 females). Many patients were not aware of the option to stop anticoagulation and had limited understanding of the decision about treatment duration. Despite the negative quality-of-life impact of clinically relevant bleeding during VTE treatment, the majority continued anticoagulation due to emotional trauma of VTE diagnosis, a perception that bleeding would be more manageable than VTE recurrence, a desire to maintain a connection to subspecialty care or non-VTE related benefits (eg, cancer diagnosis, protection from COVID-19). Patients’ decisional needs included lack of choice awareness, inadequate support for participation, lack of personalized risk stratification, and inadequate information on monitoring and managing heavy menstrual bleeding.

**Conclusion:**

Despite the impact of anticoagulant-associated bleeding on quality of life, patients preferred continuing with anticoagulation for reasons extending beyond secondary VTE prevention. Effective decision-support interventions are needed to address unmet decisional needs.

## Essentials

• Extended anticoagulant treatment is recommended after unprovoked venous thromboembolism.
• Patients who had venous thromboembolism and bleeding were interviewed about their experiences and decisional needs.
• Despite bleeding and its impact on quality of life, patients preferred continuing anticoagulation.
• Decision-support interventions are needed to address unmet decisional needs.

## 1 Introduction

Venous thromboembolism (VTE), comprising deep vein thrombosis and pulmonary embolism, is a significant cause of morbidity and mortality, but treatment is not without risk. Approximately half of VTEs are not attributable to a transient or persistent risk factor and are thus classified as unprovoked. Unprovoked VTEs carry significant risk of recurrence, with rates up to 16% at 2 years and 36% at 10 years [1]. With such a risk, major guidelines recommend considering indefinite use of anticoagulation in patients with unprovoked or weakly provoked VTE after an initial treatment of at least 3 months [2,3]. Although highly effective at preventing VTE recurrence, anticoagulation comes with a risk of bleeding. While major bleeding remains the most serious complication of anticoagulants, with an incidence of about 2% per year and a case-fatality rate of about 10%, clinically relevant nonmajor bleeding occurs 3 to 5 times more frequently than major bleeds and can be equally burdensome and costly [[4], [5], [6], [7]]. Taken together, oral anticoagulants are the most common cause of medication-related emergency hospitalizations among older adults in North America [8]. When there are pros and cons to long-term treatment, it is recommended to involve patients in decision-making. Indeed, many guidelines promote the use of shared decision-making with patients on whether to stop or continue anticoagulant treatment after the initial 3 months based on individual risks of VTE recurrence and bleeding [2,3,5]. However, they generally recommend long-term treatment in the absence of contraindications (ie, high bleeding risk) to minimize risks of recurrent VTE [2,3,5]. A 2020 systematic review and meta-analysis reported higher values patients placed on VTE risk reduction over treatment-related harms in primary VTE prophylaxis [9], a finding that mirrors survey results in the setting of secondary VTE prevention [10,11]. Similarly, in a recent qualitative study of 18 patients receiving extended anticoagulation for unprovoked VTE in the Netherlands, most patients focused on preventing VTE recurrence and were less concerned with bleeding [12]. Nonetheless, the study enrolled a low number of women, and no patient had experienced a major bleed. Hence, it is unclear how patients with unprovoked VTE consider the risks and benefits of anticoagulation after suffering a major, clinically relevant nonmajor, or minor bleeding event.

Therefore, we sought to explore experiences, values, and decision-making needs among patients who have continued anticoagulant therapy for an unprovoked VTE despite experiencing an anticoagulant-associated bleed.

## 2 Methods

### 2.1 Study design

We conducted a descriptive qualitative study using semistructured interviews. Research ethics approval was obtained from the Ottawa Health Science Network Research Ethics Board (20210620-01H). The article is reported according to the consolidated criteria for reporting qualitative research checklist [13].

### 2.2 Setting and study population

Recruitment occurred at a large academic hospital with over 16,000 patient visits for diagnosis and treatment of VTE annually (in Eastern Ontario, Canada) [14]. Eligible participants were adults (≥18 years) who experienced both bleeding (any site or severity) and unprovoked VTE within the previous 2 years. Unprovoked VTE was defined as VTE without transient or persistent provoking risk factors according to guidance by the International Society on Thrombosis and Haemostasis [15]. Exclusion criteria were inability to consent, insufficient English, Chinese, or Dutch language ability, and other factors interfering with patients’ participation in an online face to face interview.

### 2.3 Study procedures

Patients were recruited by their treating physician from a thrombosis subspecialty clinic at the hospital. To maximize the range of experiences and views, purposeful sampling was used based on sex, age, and type of bleeds [16,17]. By purposeful sampling, we aimed to include roughly as many female participants as male participants, participants in both the working-age population as well as elderly, and patients with nuisance bleeding, minor bleeding, and major bleeding, all occurring in different sites of the body. Patients who were willing to be interviewed were contacted by a research team member to arrange an online, one-to-one interview through Microsoft Teams. Interviews were conducted by 1 of 2 authors (P.W. or Y.X.) specialized in adult thrombosis medicine, ensuring no patient-physician relationship between the participant and interviewee. Patients provided informed consent before the start of the interview. After 10 interviews, data saturation was monitored to determine if any new themes were identified in the previous 3 interviews [18]. Interviews were stopped when purposeful sampling and data saturation were reached (eg, a diverse population according to previously mentioned criteria and no new coding themes appearing in the last 3 interviews). Data saturation was reached after 13 interviews, after which an extra interview was conducted where no new themes were identified.

### 2.4 Interview guide

All interviews were conducted using the interview guide, which was developed prior to the first interview (Supplementary Material). This interview guide was adapted from a prior qualitative study in the Netherlands [12]. The main questions in the original Dutch interview guide were about patients’ 1) understanding of their VTE and anticoagulant treatment and perspective on received information; 2) experience with how the actual decision between stopping and continuing anticoagulation was made; 3) perspectives on risks and benefits of extended anticoagulant treatment; and 4) considerations regarding the decision to stop or to continue anticoagulant treatment, including their decisional needs. For the present study, we included questions about their perspectives on bleeding (eg, personal experiences, impact of bleeding on daily life, and their decisions regarding anticoagulant treatment) and additional questions to evaluate their decisional needs when choosing whether to continue anticoagulation. The interviewers used a natural progression to cover the questions in the interview guide, which allowed for flexibility in following along with how the interviewee responded to the questions.

### 2.5 Statistical analysis

Interviews were audio-recorded and automatically transcribed verbatim using Microsoft Teams built-in software. Transcriptions were verified immediately after the interview. Subsequently, transcriptions were uploaded to a computer-assisted qualitative data analysis software program (NVivo version 12 Pro, 2018; QSR International Pty Ltd). Directed content analysis was used to analyze the data. Directed content analysis can be used to validate or conceptually extend an existing theoretical framework [19]. Content analysis was guided by the codes and themes identified in the previously published Dutch study [12]. These codes and themes were added into NVivo to facilitate the analysis. For findings that could not be coded under existing codes or themes, we identified new codes under new (sub)themes. The first 2 interviews were coded line-by-line independently by Y.X. and M.W. The coding of these interviews was discussed by Y.X. and M.W. to ascertain the consistency of coding and interpretation of the codes. Themes were reported by the following research objectives: 1) experiences of anticoagulant-associated bleeding; 2) reconciling competing risks of recurrent VTE and bleeding; and 3) decisional needs. All subsequent interviews were analyzed by Y.X. Themes with illustrative quotes were audited by all authors to reach a consensus.

## 3 Results

Fourteen interviews were conducted between September and December 2021. Data saturation was reached after 13 interviews. The median interview duration was 43 minutes (range, 21-86). The median age of the interviewed patients was 53 years (range, 41-69); 9 participants identified as female and 5 as male (see Table 1). One participant was Asian, and all other participants were Caucasian. All patients had continued anticoagulation after the initial treatment period.

**Table 1:** Characteristics of the participants.

Patient	Age	Sex	Level of education	Index event	Time between VTE and interview	Time between bleeding and interview	Type of bleeding
1	49	F	PhD	Proximal DVT	6 mo	Does not recall	Menorrhagia
2	41	M	High school	Low-risk PE	10 mo	Does not recall	Bruising
3	43	M	Master’s	Proximal DVT	14 mo	10 mo	Hematochezia
4	48	M	NA	Intermediate risk PE	12 mo	10 mo	Hemoptysis, anemia with transfusion dependence
5	69	M	Master of Education	Proximal DVT	6 y	3 y	Hematuria, ICH
6	62	M	Military college	Proximal DVT	12 y	10 y	GI bleed, ICH
7	46	F	Master’s	Intermediate risk PE	9 mo	8 mo	Menorrhagia
8	53	F	High school	Low-risk PE	6 mo	5 mo	Menorrhagia
9	67	F	Postsecondary	Recurrent distal DVT	23 mo	22 mo	Menorrhagia
10	53	F	Master’s	Intermediate risk PE	5 y	14 mo	Menorrhagia, bruising
11	57	F	High school	Standard risk PE	12 mo	10 mo	Rectal bleeding
12	63	F	College	Proximal DVT	8 mo	4 mo	Epistaxis
13	47	F	College	Low-risk PE	17 mo	17 mo (bleeding precedes VTE diagnosis)	Hematoma at injection sites; menorrhagia
14	54	M	Postsecondary	PE	3 y	2.5 y	Hemoptysis

Due to limited variation among participants, disclosing race information could lead to identification of the individual subjects. Therefore, this information is not shown in Table 1.

DVT, deep venous thrombosis; GI, gastrointestinal; ICH, intracranial hemorrhage; PE, pulmonary embolism; VTE, venous thromboembolism.

### 3.1 Experiences of anticoagulant-related hemorrhage

**Figure 1:**
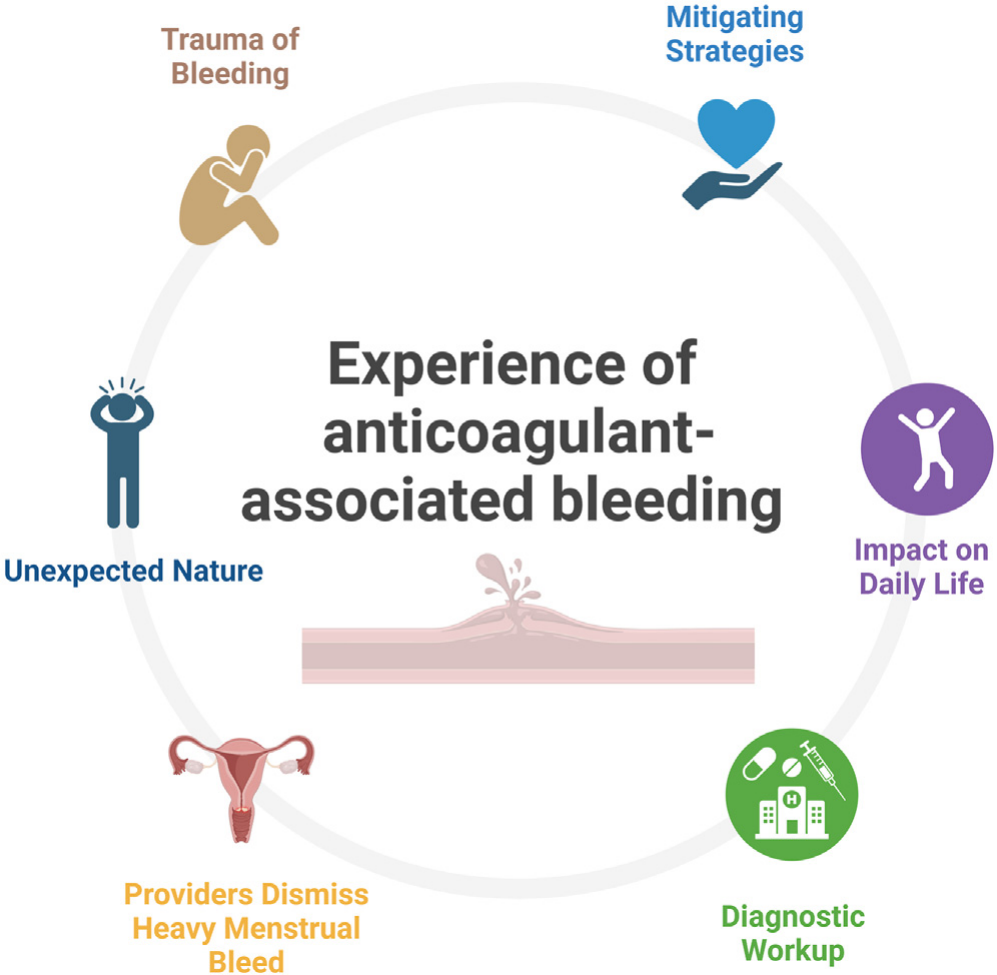
Patients’ experience of anticoagulant-associated bleeding with main themes identified.

Themes within patients’ experiences regarding anticoagulant-related bleeding included the bleeding’s impact on daily life, unexpected nature of bleeding, provider dismissiveness with heavy menstrual bleeding among young women, and diagnostic work-up following bleeding (Figure 1).

Patients who suffered a bleeding event, regardless of severity based on clinical research nomenclature, described the theme of tangible impact of these bleeding episodes on their activities of daily living and employment.

*“I basically had planned on retiring next May but I'm basically retired now. If I had been still working, it would have been very uncomfortable and I wouldn't be able to [work] because, I mean, what would I do if I was working and [the rectal bleed] happens? Anyways, I just left [the job]. I stay retired [and] I'm home most of the day, which is nice, so I'm able to not have to worry about being out other than to, you know, go for groceries or that kind of thing. I really don't leave my house a lot except to take the girls back and forth to school and all that stuff.”*

(Patient 11)

Another theme was provider dismissiveness with heavy menstrual bleeding among young women on anticoagulation.

***Patient:****The doctor goes, “how are things on the blood thinners?” It's like, Well, they're fine. Except the period has gotten a lot heavier. And I think he's used to dealing with people in an age group that aren't having these problems, so it to me felt a little fluffed off like, “Oh well, it's just some more bleeding. You're used to that anyway.” At least that’s how I felt. But to me it's time consuming: it's laundry; it's, you know, cleaning up; it’s affecting my life in a way that I don't want it to be affected. I have other things to do every day. I don't have time to deal with that.*

(Patient 13)

The effect of anticoagulant-associated bleeding, however, was not universal; several patients did not find the increased bleeding impactful on their lives, while others were able to find alternative strategies to mitigate the sequelae of anticoagulant-associated bleeding (Table 2).

**Table 2:** Patients’ experiences on having bleeding.

Themes	Additional quotes
Impact on daily life	*I get all the blood and then I have to wipe myself off. It’s the fact that you know you got to go to the washroom, you know what’s gonna happen and it’s like, “Oh, I hope it’s not too bad this time. I hope it’s not too bad.” You know, it’s seeing those blood clots [in the stool] and I’m really glad that I don’t have anybody around me to know that that kind of thing is happening, it’s horrible. (Patient 11)*
	*It wasn't very impactful. I mean as a younger person, I did have a much higher level of bleeding actually. The original reason that I didn’t go on birth control was because I was trying another medication for a few years as a late teenage early in my 20s that was no longer functioning without having to double the dose. (Patient 10)*
Bleeding was unexpected	*It could make it worse, but it’s not a cause of [bleeding], so they decided to check to see what was causing it because I’m a little old for that…what 66 years old? So it’s like a this is long gone; I don’t need this. Like it’s not so much quality of life. It’s like I’m done with this [menstrual bleeding] … I don’t need this anymore. (Patient 9)*
Bleeding led to a diagnostic work-up	*Well, it at least it gave me answers. Because of the blood clots, they wanted me to do all of these tests to determine that it wasn’t cancer that was causing the clot [and] bleeding. And I’m not one to complain very much. If it happens, it happens as long as there’s a light at the end of the tunnel. (Patient 10)*
Trauma of bleeding	*The neurosurgeon said, Oh yeah, it’s nothing. We drill 2, I don’t know, quarter inch holes in your in your skull and he says it’s very small and then I’m looking at the end of my baby finger and I said that’s not small, no. You know, like who wants that. But anyway, that’s what he said. (Patient 6)*
Finding alternate strategies to mitigate	*I went back from maybe a 3 day [period of menstruation] to a 5 day [period]. It wasn’t heavier, it just I find the length of time was a little longer sometimes – it may have added a day or a second day, but in terms of the bleeding itself, there wasn’t any major increase, and why I actually got to the hysterectomy was not just based on the medication – I did have a fibroid as well that had been identified in 2016. (Patient 10)*

Some interviewees noted unanticipated benefits arising from investigations undertaken during management of anticoagulant-associated bleeding, including the detection of occult malignancies.

*“There has been another benefit to the blood thinner. Because in a few years back, I got severe anemia. And then I had a bleed and I had H pylori in the stomach and I had a cancerous polyps in my colon. And I credit the actual blood thinners for early detection of that, those problems.”*

(Patient 6)

Relevant quotes about additional themes identified, including trauma of bleeding and alternate strategies to mitigate bleeding, can be found in Table 2.

### 3.2 Reconciling competing risks of recurrent VTE and bleeding

When faced with the decision to continue or stop anticoagulation after completing the primary treatment of an unprovoked VTE, patients had wide-ranging experiences, from absence of shared decision-making to being fully informed and involved in the decision.

*“At no point in time did they say, we will keep you on [anticoagulants] for this amount of time, and then take you off it and monitor you closely [off anticoagulants]. They don't seem to have that.”*

(Patient 6)

Patients also had varying perspectives on their desired degree of involvement in decision-making related to anticoagulant duration in unprovoked VTE. Some relied strongly on their physician’s recommendations, others preferred to decide for themselves based on information offered.

*“I guess I just felt comfortable enough to have faith in the recommendation of the specialist. The person is this specialist, I assume they're gonna take my health and my best interest [into account as much] as possible and give me the solutions that are available for me.”*

(Patient 10)

Additional quotes about themes of comfort with going against advice and suggestions for shared decision-making can be found in the Supplementary Material.

**Figure 2:**
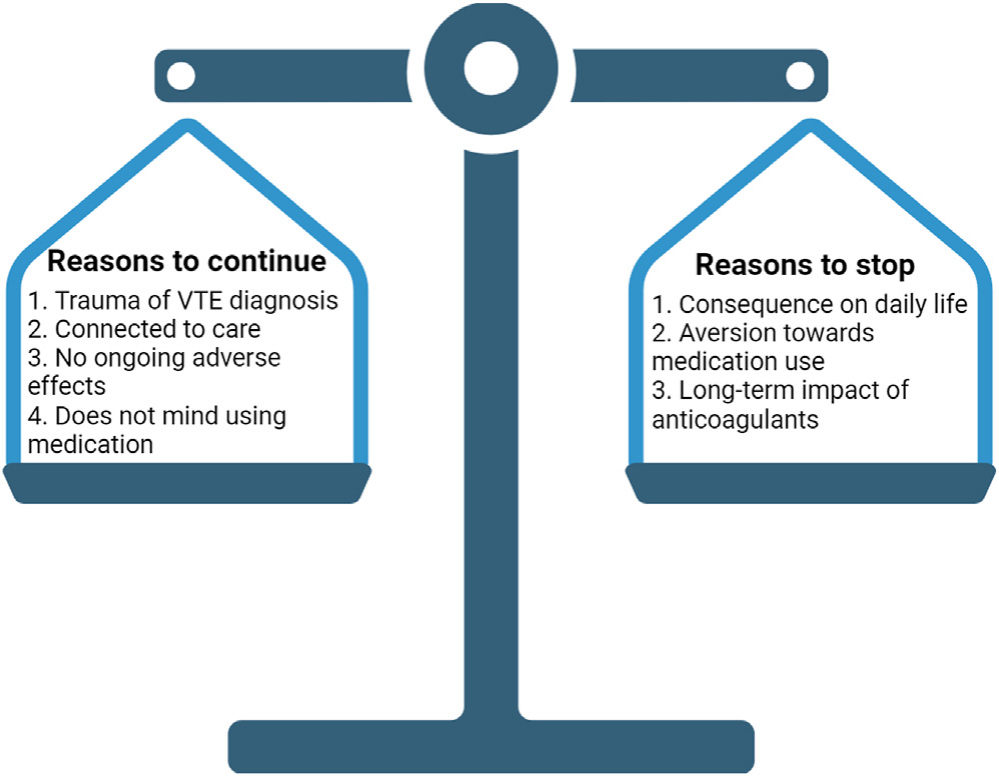
Patients’ identified reasons to continue or stop anticoagulation after suffering an anticoagulant-associated bleed during unprovoked venous thromboembolism (VTE) treatment.

Despite describing several factors that favored stopping anticoagulation due to bleeding complications (Figure 2 and Table 3), patients’ decisions to continue treatment despite suffering ongoing bleeding complications centered around 4 themes: trauma of initial VTE diagnosis, a perception that bleeding would be more manageable than VTE recurrence, being connected to subspecialty care, and non-VTE related benefits of anticoagulation (Table 3).

**Table 3:** Factors influencing the decision to stop or continue anticoagulation.

In favor of continuing	In favor of stopping
No ongoing adverse effects *I think it’s better if I continue, because it doesn’t bother me. I’m OK right now, I don’t have any blood and my nose is not bleeding anymore so I’m OK. It’s just one pill; I don’t mind taking it. I don’t have any other reaction. I don’t have any anything else. It’s just like if I was taking a Tylenol or something. There’s no reaction.* (Patient 12)	Consequence of therapy on daily life *I mean I play relatively decent caliber hockey, but I also now realize I don’t go as hard to the boards. I don’t like. I would never stop in front of shots like you know, being aware of the bruising and whatnot so it’s slightly changed my perspective of how hard I try.* (Patient 2) *I am walking now back walking 4 blocks without any problem and I’m feeling much better but the bleeding just never seems to go away. It’s so it’s very frustrating, because the amount of blood that I lose, I mean, a girl who had a menstrual cycle would definitely lose that much blood all the time. It’s a lot. And then of course sometimes when I just passed gas, it will come out too, so it's been really frustrating.* (Patient 11)
Does not mind using medication *“The fact of how well I had responded, you know? I was hopeful [that anticoagulation can be stopped]. Everyone’s hopeful. I’m not a person who likes to take medication in general. I always avoid them, try to find alternative solutions before going to medication. So I was disappointed that I would have to continue because you know, it’s not my thing to just take medication. But at the same time, the reaction that I had in in those 6 months was very positive, which left me thinking continuing on the medication and knowing that you know they [thrombosis unit] are very open. If anything changes. If you’re not comfortable, if you’re worried about anything, give us a call*.*”* (Patient 10)	Aversion toward (unnecessary) medicine use *The ultrasound saying exactly where [the clot] was, and also the blood test said a lot, so all these are important because it’s not just out of the blue like this that you have to continue [anticoagulants], it has to have some proof – like the tests were saying.* (Patient 12)
Therapy works and protects *“Would I have been happy to be on treatment? Not really, but I guess the benefit so far outweigh the hassle. […] You know ‘cause I would certainly see warfarin as being a hassle. But if it provided the benefit and the benefit would be to mitigate the risk of development of future blood clots, then it would be worth it.”* (Patient 5)	Long-term impact of anticoagulant therapy *You know the long-term effects; I’m still young enough that we could be talking at, say, 40 years, and what that would look like in the long term. At this point in time I'm not so concerned with the fear of the blood clot coming back. At this point, things are going well; should they not go well, that's another thing. I think if something changes and I have to change the medication, what would that mean for me? Those are the 2 things that I would think would be most on my mind.* (Patient 10)
Trauma of VTE diagnosis “*The blood thinner doesn't frighten me. The clot frightens me if* I'm *not on the blood thinner.*” (Patient 4) *It was treated very quickly with me, I had a lot of positive reaction to the medication, so I never really thought of the serious consequences of it, because things worked out quickly for me. The result was positive. But knowing that…when I had heard of this person passing away, who was similar in age to me and that I knew personally, it kind of brought home to me. Wow, OK, it could have been much worse than my situation, and that having a blood clot can have very serious consequences if it's not diagnosed and found before it actually moves further along.* (Patient 10) See additional in-text quotes	Fear of bleeding *I guess the way I look at it, if a blood clot comes back then I'm back on thinners anyways, right? But with a bleed, um, it's probably more serious. I don't know. It's more serious, can be more serious, right? Not necessarily, though. So I guess I don't really have the kind of feeling…like it would be safer for me [to stop] if I was really, really prone to bleeding. To go off the thinners and deal with the blood clots.* (Patient 4)
Connected to care *“But having the blood thinners is very reassuring, knowing that you already have those in your body and if should something happen that you already are taking that medication. That you can get connected; you know to someone at the thrombosis clinic if you feel that there's something going on with your health. So it does give me some comfort knowing that I am on [anticoagulants] should there be another event*.*”* (Patient 10)	
Perceived benefit for non-VTE related conditions *I personally feel that there are some advantages to blood thinners, associated with the heart, a protection. That's sort of, I believe, that in ways I think there are you know, I don't have the details, but I believe there is some advantages there.* *“When I had my last discussion, I prioritized my mental well-being [by continuing hormone replacement therapy, thus remaining on anticoagulation] because I feel like to me that was important, but without understanding that there might be consequences to continuing this pill in the longer term. If the consequences you are talking about 5% bump in risk, I would probably still take the risk, but if the risk grows over time and makes it likely that I might have a bleed out, I might not feel the same way.”* (Patient 7)	

Initial diagnosis of VTE for many patients included in the study came as a surprise, as they did not believe they fit the profile of a *typical* VTE patient. Several patients attributed their age or physical activity levels as factors protective against the development of VTE and were therefore surprised when investigations of their presenting symptoms led to a final diagnosis of VTE. Therefore, the unexpectedness of VTE diagnosis influenced their values and preferences pertaining to balancing risks of recurrent VTE and major bleeding. In addition, the experiences that led to the diagnosis of VTE, specifically among patients with pulmonary embolism, were traumatic. This trauma of VTE diagnosis is in part attributable to inadequate information that is provided to patients at the time of their VTE diagnosis and led to some interviewees to seek mental health support.

*“[The emergency physician] was like, ‘This pill will keep you alive tonight and our team up in the thrombosis clinic will be in touch first thing tomorrow; expect them to call you.’ And then he said good luck to you. [Then] they're like: You can leave. It was like 1:00 o'clock in the morning …and I was like, Whoa. Pill’s going to keep me alive for the night, and I think that really marked me and I ended up calling my family doctor the next morning, because I was completely and utterly overwhelmed and had zero information of what I could expect."*

(Patient 7)

Participants’ assessment of predictability and the capacity to manage recurrent VTE compared with major bleeding was a crucial component of their decision-making on anticoagulant duration.

*“I would definitely choose [to have] the least chance for me to have the blood clot, because the bleeding is kind of manageable, whereas the blood clot is a sneaker and, that's really scary.”*

(Patient 11)

In addition, participants placed weight on being connected to a specialty clinic as a reason to continue treatment.

*“I guess it depends what other benefits I'm getting from being on them, like I don't know if once blood thinners are stopped, do you come back in six months and get checked just to make sure your levels are OK. I think I'd be uncomfortable stopping and not having a follow-up.”*

(Patient 12)

Interviewees reported aspects of anticoagulant treatment that are indirectly related to prevention of recurrent VTE itself:

*“These studies they've done on COVID that says people on blood thinners are less likely to have issues with COVID. I think I read that somewhere – something about blood thinners and people that are on them*.”

(Patient 9)

### 3.3 Decisional needs

Themes identified in our study aligned with aspects of the Ottawa Decisional Support Framework, specifically within the Decisional Need domain of the Framework (Table 4). Some interviewees who were aware of the decision to stop or continue anticoagulants reported decisional conflict pertaining to having to choose between 2 potentially life-threatening outcomes:

*“So I wasn't on any blood thinners at all [after the bleed], which sort of frightened me a bit. But I knew it was either I took the pill, or I didn't take the pill and got a clot. So, I was sort of between, twixt in between.”*

(Patient 4)

**Table 4:** Decisional needs of patients facing the decision to stop or continue anticoagulation after the initial treatment for venous thromboembolism.

ODSF decisional needs	Themes	Quotes
1.1 Difficult decisional type and timing	Competing consequences of thrombosis and bleeding	*Risking another bleed that could put you in the hospital. I guess that would be where, I guess you'd say, get off the blood thinners if you can, but then, who's gonna watch and make sure that I don't get another clot?* (Patient 11)
	Impact of future health changes on anticoagulant options	*“I think if something changes and I have to change the medication, what would that mean for me?”* (Patient 10)
1.2 Unreceptive decisional stage	Feeling very vulnerable	*When you're diagnosed with something that's potentially life-threatening no matter what it is, you're in a very vulnerable state. You're in a fragile state, emotionally and mentally, and trying to figure out what it all means.* (Patient 7)
1.3 Decisional conflict	Feeling uncertain about which option to choose	*So I wasn't on any blood thinners at all [after the bleed], which sort of frighten me a bit. But I knew it was either I took the pill, or I didn't take the pill and got a clot. So I was sort of between, twixt in between. (Patient 4)*
		*Having another blood clot but risking another bleed that could put you in the hospital. I guess that would be where, I guess you'd say, get off the blood thinners if you can, but then, who's gonna watch and make sure that I don't get another clot?* (Patient 11)
1.4 Inadequate knowledge	Need for personalized risk information	*I would want to hear what my clinicians are telling me and warning me about the risks and be able to make a good, informed decision about my body and which risks I'm most concerned about. But I cannot do that if I'm not equipped with a the best possible information and an honest assessment of what those risks are. And I I'm clearly not qualified to determine essentially what those risks are.* (Patient 7)
	Lack of awareness on consequences of bleeding	*I think it's better if I continue, because it doesn't bother me. I'm OK right now, I don't have any blood and my nose is not bleeding anymore so I'm OK. It's just one pill; I don't mind taking it. I don't have any other reaction. I don't have any anything else. It's just like if I was taking a Tylenol or something. There's no reaction. I didn't have a lot of complication; it doesn't matter if I take it or not, I don't see the difference, why not take it?* (Patient 12)
	Mistaking venous and arterial thrombosis	*I was scared. I was scared because my understanding is a blood clot like that [venous thrombosis] could cause you to have a stroke. And well, it could kill you*. (Patient 11)
		*You know, you hear so many things about blood clots traveling, [venous] blood clots can cause a stroke. Because of the history in my family of heart issues, stroke issues, I didn't want to take the chance.* (Patient 9)
	Long-term impact of anticoagulant therapy	**Interviewer**: *Is there anything that you want to discuss or that you think are relevant for patients who are starting on anticoagulant treatments that we haven't covered.*
		**Patient**: *You know the long-term effects; I’m still young enough that we could be talking at, say, 40 years, and what that would look like in the long term. At this point in time I'm not so concerned with the fear of the blood clot coming back.*
1.5 Unrealistic expectations	Unfounded worry about a residual thrombus	*I know [the clot is] still there. It's not gone yet, so I'm afraid that it would get bigger again or move in my body. That's what I worry about*. (Patient 12)
	Unrealistic fear of bleeding	*I guess the way I look at it, if a blood clot comes back then I'm back on thinners anyways, right? But with a bleed, um, it's probably more serious. I don't know. It's more serious, can be more serious, right? Not necessarily, though. So I guess I don't really have the kind of feeling…like it would be safer for me [to stop] if I was really, really prone to bleeding.* (Patient 4)
1.6 Unclear values	Therapy feels insufficient	*There was a recommendation that I could go to 2.5 milligrams twice a day as opposed to the 5 [milligrams]. That being said, as you mentioned, there is some hesitancy. Sure, if we're going to have another blood clot, you know the type of work that I do - I do stationary [work] for a large part of the day at different moments, so I wasn't fully comfortable changing and at that time the specialist that told me, “You know, 2.5…5…they're very similar.” So he decided to leave me on the 5 milligrams.* (Patient 10)
	Connected to care providers	*I guess it depends what other benefits I'm getting from being on [anticoagulants], like I don't know if once blood thinners are stopped, do you come back in 6 months and get checked just to make sure your levels are OK. I think I'd be uncomfortable stopping and not having a follow-up.* (Patient 13)
1.7 Inadequate support and resources to make/implement the decision		
a. Lack of information on options from healthcare professionals	Insufficient SDM/explanation re: bleeding risks	*And so I said, I've got this balance here and if it means taking this pill longer, I'm OK with that. What didn't come out or what I may not have heard is that there are perhaps risks that go up with continuing this pill for any length of time beyond that 3 to 6 month period.* (Patient 7)
	No options discussed	*But in my recollection, there was no option and I can remember talking to him well. You know, they said you'll need to be on blood thinners and I said, well, for how long and their answer was, well, you can't really go off it. … But I was left with no option but to go on blood thinners.* (Patient 6)
		*No, I wasn't presented with an option to say,* “*OK, this is the alternative solution if you don't stay on blood thinners*.” *It was recommended that I continue*. (Patient 10)
d. Pressure from others	Feeling pressure to take anticoagulants	*Now I did always try and push them to reduce the amount. And up till just recently with doctor [name redacted], it’s the first time I've seen him and up until him they wouldn't reduce it. They didn't. They felt you can't. I said, can I break the pills in half and try half and then the doctors weren't buying that but I convinced Dr. [name redacted]. He let me drop it to half the dose I'm taking*. (Patient 6)
e. Inadequate experience, self-efficacy	Deferring to specialist expertise	*The person is this specialist, I assume they're gonna take my health and my best interest [into account as much] as possible and give me the solutions that are available for me*. (Patient 10)
f. Inadequate emotional support, advice, and instrumental help	Virtual care interferes with receiving adequate support	*I think a phone call is very depersonalizing; The sense [I had] is that the doctors are making a bunch of calls and you're on the list of calls to be made, which is fundamentally a different experience than when you're sitting either face to face where they might take a look at your face, whether in-person or not, and see just how friggin scared you are. And you can't necessarily convey on the phone unless you start crying – which is what I fundamentally did with my family doctor. But when I have a stranger calling me, I don’t want to say I felt like a number, but I felt like I was one of a number of calls that needed to be made, so whereas I would have advocated for myself through asking questions if I had been in-person, on the phone I felt like “Well. This person needs to get on to the next call.”* (Patient 7)
	Limited access to anticoagulation blood monitoring (warfarin)	*When you say when you're talking about the treatments, they have to be doable for people. And if I'm in the country and I'm having a snow storm and I can't get out, sure, it's inconvenient, but it's also affecting my treatment if I can't get those [INR] numbers to the doctor, right?* (Patient 13)
	Limited funding for medications	*It's 30 bucks like it's not even it doesn't even impact in my life at all, but somebody who might be in a different financial situation might be like. OK, well should I weigh the costs of this?* (Patient 2)
	Emotional distress	*I actually got psychological help as I found the experience in the end to be far more traumatic than I expected it to be. Because for weeks, I was worried I wouldn't wake up in the morning, so I would be up at night hoping that I wouldn't fall asleep so that I wouldn't, you know, be worried about not waking up.* (Patient 7)
	Trauma of bleeding: impacts decision-making on willingness to participate in SDM	*The clots are like, crap. I got a clot again…off I go to the hospital. You know when they check? Yeah yeah, that's what it is and they deal with the clot, right? You know, I learned to feel what a clot felt like and it always occurred in the same spot. Right, so you know, it was an inconvenience for me at the time, right? Yeah, it's serious enough. We know we know that we know it's serious, but it was inconvenient did not have an impact on my life. The brain bleed was a blip in time. And it was very, very traumatic. But it did impact my life. I remember going to write my will en route to the hospital.* (Patient 5)
	Virtual care limits access to meeting my information needs.	*I wish they had given me more [information]. That's the hard part when we are doing [consults] over the phone. Doctor calls you and you don’t always get the opportunity to ask what you need to ask. Because they're on a schedule, which I understand and everything, but if you’re in the office, you might get the chance to just say “oh, by the way, I was thinking” and then get a chance to speak. So yeah, it's been different being doctored over the phone rather than being doctored in-person.* (Patient 11)
	Suggested requirements for SDM	*I might make the exact same decision and I might not, but I think that the better informed your patients are, it will afford them to make better decisions and feel comfortable with the decision, whatever the consequences to them. they want information, they want knowledge, and they know that that knowledge is that power, but knowledge will only take you so far because that you also have feelings and instincts about what's right for you and for a patient you'll have some that will focus almost exclusively on the knowledge, and others who will be far more on the instinct. [They] may not be making the choices that you would want them to be making in that regard, but I guarantee you that they will make the wrong choices every time if they have to be the ones to diagnose themselves and understand their diagnosis and make decisions without information, and so I would say we can never underestimate the power of good, solid, reliable information, because I do believe it will make a difference to people.* (Patient 7)
	Provider dismissive of heavier menstrual bleeding	*Except the period has gotten a lot heavier. And I think he's used to dealing with people in an age group that aren't having these problems, so it to me felt a little fluffed off like, “Oh well, it's just some more bleeding. You're used to that anyway.” At least that’s how I felt. But to me it's time consuming: it's laundry; it's, you know, cleaning up; it’s affecting my life in a way that I don't want it to be affected. I have other things to do every day. I don't have time to deal with that.* (Patient 13)
1.8 Personal and clinical needs	Time to discuss treatment options	*If somebody had taken the time to explain that in a bit more detail, not saying I would have been necessarily less afraid, but the cognitive part of me would have been more satisfied*. (Patient 11)

SDM, shared decision-making.

Furthermore, important areas in which patients lacked information were identified, including long-term impact of anticoagulation, personal risk information, unawareness of bleeding complications, and confusing venous and arterial thrombosis. Some patients had unrealistic expectations, including that bleeding would not impact their lives much, that the clot would remain in its place, or that a low dose of anticoagulation would not be effective in preventing another VTE. Other identified decisional needs include lack of emotional support and resources. Moreover, several interviewees felt that the quality of information exchange to support decision-making pertaining to VTE treatment was compounded by virtual care in the context of COVID-19 pandemic.

## 4 Discussion

This qualitative study provides important insights into values, preferences, and unaddressed decisional needs pertaining to duration of extended anticoagulation among patients who have continued treatment for an unprovoked VTE after suffering an anticoagulant-associated bleed. Although bleeding during treatment of unprovoked VTE had important implications for patients’ quality of life, the majority of patients identified reasons to continue anticoagulants. This was often related to traumatic experiences at the time of their VTE diagnosis, the perception that management of bleeding complications is easier, a desire to stay connected to care, and other non-VTE-related benefits of anticoagulation. We identified several unmet decisional needs, including lack of choice awareness, inadequate support for patient participation, lack of personalized risk information, and inadequate information on the identification and management of VTE and heavy menstrual bleeding.

While several studies have elicited patient perspectives, lived experiences, and psychosocial impact of VTE at the time of diagnosis and longitudinal follow-up [[20], [21], [22]], our study adds perspectives of patients who must navigate the competing risks of recurrent VTE and anticoagulant-associated bleeding after suffering both thromboembolic and hemorrhagic complications. Studies in patients using antithrombotic treatment for indications other than VTE found that a significant decrease in quality of life was observed for even minor bleeding events [23]. In this study of patients with VTE, we identified occupational, social, and psychological impacts associated with not only participants who suffered major bleeding but equally among those who experienced events classifiable as clinically relevant nonmajor or minor bleeding. This quality-of-life burden is especially notable among individuals who suffer mucocutaneous bleeds, such as those involving the gastrointestinal or genitourinary tract, and those whose symptoms tend to be protracted. The majority of female participants experienced menorrhagia. Their experiences will likely be different from those of patients with acute major bleeding events, who represented only a small proportion of our study population. We hypothesize that for patients who had major bleeding, fear of long-term complications of major bleeding (eg, physical impairment after intracerebral bleeding) may be more influential than the risk of nuisance bleeding in day-to-day life. However, the impact of menorrhagia may be similar to other frequently recurring bleeding events (eg, rectal bleeding). It is important to acknowledge that heavy menstrual bleeding is frequently encountered by women on anticoagulation worldwide and may have a strong impact on their quality of life. Despite the considerable impact of such bleeds on quality-of-life reported by our participants and described in the recently published literature [24,25], we found that patients who report such events face barriers to having their symptoms recognized as adverse events by their providers. In turn, this may contribute to patients attaching less importance to bleeding than thrombosis. This highlights the urgent need for thrombosis providers to be aware of chronic, low-grade bleeds associated with anticoagulants, to actively assess for them during longitudinal follow-up, and to work collaboratively with other disciplines (eg, gastroenterology or gynecology) to mitigate these adverse effects in a timely fashion.

Despite the personal and professional toll of anticoagulant-associated bleeds reported by participants in the study, we observed complex processes of reconciling risks of recurrent VTE and bleeding that ultimately influenced their decision to continue anticoagulation. Despite major and clinically relevant nonmajor bleeds having a higher or similar rate of case fatality compared with recurrent VTE [1,26,27], we found that patients often placed more importance on the possibility of recurrent VTE than on their bleeding experiences. These findings seem consistent with what has been described in previous studies as “thromboneurosis”: heightened awareness of the possibility of having recurrent VTE, worry about potential recurrent VTE not being diagnosed timely, and fear of potential consequences of VTE [12,28]. Consistent with a recently published qualitative study that highlighted the imbalance of fear over reassurance in the emergency department at VTE diagnosis [21], several interviewees in our study described the initial anxiety associated with VTE diagnosis that stemmed from their communication with providers. Ongoing and untreated psychological distress experienced in a large proportion of patients following a VTE diagnosis [29], in turn, influences patients’ ability to participate in shared decision-making for anticoagulation duration after a first unprovoked VTE. Furthermore, participants in our study were concerned about losing access to a specialty care clinic and its resources by stopping anticoagulation. These findings, therefore, call for rigorous evaluation of psychological support and reassurance to patients at the time of VTE diagnosis, as well as the role of care continuity among patients who stopped anticoagulation.

Risk prediction tools may be used to provide individual risks of recurrent VTE and bleeding and to help facilitate discussions of net clinical benefit [30,31]. In our study, some patients expressed a desire to know what their personal risk of recurrent VTE or bleeding would be; low estimated risks of recurrence may provide reassurance when stopping therapy. In addition, short-term clinical follow-up after oral anticoagulant cessation may provide additional assurance and be an important element of care continuity, an area of need identified in our study.

Our findings revealed several unmet decisional needs. First, a considerable proportion of patients were not aware that there was a choice to be made. Choice awareness is essential as a first step in shared decision-making [32]. Some did not consider this problematic, as they stated that they prefer just to follow their physician’s advice. This may result from a feeling of being unable to participate rather than an unwillingness to participate [33]. Patients need support to make informed decisions, which requires having psychological and emotional bandwidth for these intricate decisions [33]. In addition to power, knowledge is crucial to facilitate shared decision-making [33]: some patients lacked information in key areas and had important misunderstandings. For example, patients expressed a need for more information regarding long-term effects of anticoagulation. Misunderstandings include, among others, fear of stroke due to mistaking venous and arterial thrombosis. Information to bridge these knowledge gaps and misunderstandings may be incorporated into patient education tools as well as in educational activities for physicians. Patient decision aids and coaching are effective interventions to improve patient knowledge, set more realistic expectations, and support patient involvement in decision-making [34,35]. To improve patient-centered care, a first step could be to measure quality-of-life outcomes in a standardized way in clinical practice, for example, by using the International Consortium for Health Outcomes Measurement’s set of patient-centered outcomes for VTE [36]. This may help to identify patients’ needs and, at a later stage, measure the effect of yet-to-be-developed decision-support tools. Our study’s findings may help to further update patient-centered outcome measures and decision-support tools.

As this study was conducted during the COVID-19 pandemic, many patients’ follow-up appointments were by phone rather than in-person. These phone calls did not meet the needs of some patients, demonstrating that in-person communication is very important for many patients, both to receive information and to ask questions. Whether video calls instead of phone calls may provide similar value to replace in-person visits to facilitate shared decision-making with patients remains an important area of exploration.

This study provides rich and detailed data on perspectives of patients who experienced both VTE and bleeding, a population that was thus far underrepresented in qualitative studies. By targeting this specific population, valuable insights into how patients experience bleeding were obtained. We used several strategies, including group debriefing, member-checking, and triangulation, in non-VTE settings to ensure the trustworthiness of our findings [37]. Transferability of findings was improved by providing an extensive description of participants and setting [37]. To increase confirmability of the findings, interviews were recorded, automatic verbatim transcriptions were immediately verified after the interviews, and all findings were audited by all authors to reach a consensus [37]. The *a priori* plan regarding purposeful sampling and data saturation was achieved, causing the data to be rich and relevant to our review question. A wide range of experiences was captured of patients with different severity and sites of bleeding, male as well as female participants, and participants of different ages. Due to limited variation among participants, disclosing race information could lead to identification of the individual subjects. Given that we only interviewed patients who continued anticoagulation, further research should be conducted with individuals with unprovoked VTE who chose to stop treatment after bleeding, as well as individuals with provoked VTE, as they may have different perspectives than those who opted to continue. However, it is important to note that the interviewed patients also mentioned a wide range of disadvantages of continuing treatment, indicating that they carefully weighed their options. Moreover, it was observed that some patients with unprovoked VTE considered their own VTE to be provoked as well, through factors such as lack of exercise.

## 5 Conclusion

Despite the impact of bleeding on quality of life, most patients with unprovoked VTE and bleeding continue anticoagulation. In addition to minimizing the risk of VTE recurrence due to the traumatic nature of its diagnosis, our participants identified other benefits of continuing anticoagulation that contributed to their continued use of anticoagulants. Unmet decisional needs that could be addressed were lack of choice awareness, limited support for patient participation, inadequate personalized risk assessment, and insufficient information on heavy menstrual bleeding. Effective interventions that could address these decisional needs are patient decision aids and decision coaching. Addressing these needs will improve patient-centered management of VTE.
