# Supplementary material for: Qualitative experiences, values, and decisional needs of patients with unprovoked venous thromboembolism who suffer bleeding—“This pill will keep you alive tonight”
Source: Res Pract Thromb Haemost. 2024 Mar 1;8(2):102360. doi: 10.1016/j.rpth.2024.102360 (PMC10978529; doi:10.1016/j.rpth.2024.102360)
Supplement: Supplementary Material [file mmc1.docx]

## Supplementary appendix

1. Interview guide
2. Supplemental Table

## Appendix 1. Interview guide

*(brief introduction about study aims)*

We are meeting with you because you were diagnosed with a pulmonary embolism (PE), deep venous thrombosis (DVT) or both. We also are interviewing people who have had bleeding on blood thinners. Even if you did not have a bleed we will ask you questions about this and we will be recording and later writing down your experiences and answers. The story we write for research purposes will not mention your name, age, address or any personal identifiers

*Level of understanding, knowledge of venous thromboembolism (VTE) and anticoagulant treatment and perspective on received information*

| VTE and bleeding | - What is your understanding of how the blood clot affects your current and future health? - What is your understanding of how the bleed you had or could have would affect your current and future health? Would you consider the bleed you had a major bleed, a minor bleed or a nuisance bleed? Why? - Which was/would be worse, the original blood clot or the bleed? - What had/would have the most impact on your life: VTE, or bleeding? |
| --- | --- |
| Anticoagulation | - What is your understanding of treatment for your current and future health? |
| Patient education | - What information and education did you receive about VTE and anticoagulation and from whom? - Did you feel that you had enough information about the treatment options? - Was there other information you would have liked to have known? |

*How the decision between stopping and continuing anticoagulation was made*

| Deciding on treatment duration after the initial treatment | - Were you aware there is a choice between continuing or discontinuing anticoagulation? - Did you feel involved when the decision was made? - Did your physician ask what you would prefer? - Are you satisfied with the decision made? |
| --- | --- |
| Decision making needs | - Tell me about when the decision to stop or continue anticoagulation was made? (then prompts are below) - How did you feel when the decision to stop or continue anticoagulation? - What made this decision difficult/easy for you? - When considering your options, did you worry about the consequences? - Did you feel ready and able to participate in the decision making process? - Was it clear to you what was most important to you? - What would have helped you in this decision? (choice awareness / extra information on risks and benefits, or how others decided / support from others / guidance / help to identify what option best suits your personal preferences) - Did you feel comfortable to discuss what was important to you with your physician? - Do you feel the physician provided the right amount of information to help you understand the decision that needed to be made? Too much/too little? - Did your physician find out about your preferences when it comes to stopping or continuing blood thinners? What do you wish they had known? - What was your preferred decisional role when deciding to stop or continue anticoagulation? (decide for yourself / physician decides / shared decision-making) |

*Perspective on risks and benefits of extended anticoagulant treatment*

| Anticoagulation | - When the decision was made to continue using anticoagulant medication, were you more comfortable than when it was started? - Is using anticoagulant medication having an impact on your life? - Have you experience and nuisance bleeding or other side-effects? |
| --- | --- |
| Opinion on extended anticoagulation | - What do you think are advantages of extended treatment? - What do you think are disadvantages of extended treatment? - Which advantages and disadvantages are most important to you and why? - What is your opinion about discontinuing anticoagulation after the initial 3 to 6 months of treatment? - (If on extended anticoagulation) What do you think might happen if you were to stop blood thinners? How/where did you find out about this? - (If no longer on extended anticoagulation) What do you think might happen if you were to go back on blood thinners? How/where did you find out about this? |
| Risk assessment | - How do you feel about your understanding of risks and benefits of extended anticoagulation? - What do you think is your risk of recurrent VTE over the first year and over the next 5 years? - What do you think is your risk is of severe bleeding over the first year and over the next 5 years of anticoagulant treatment? - How did you learn about your risks of another blood clot if blood thinners are stopped? Of a severe bleeding on blood thinners? (e.g., thrombosis clinicians, internet, family/friends?) |
| Risk perception | - Are you afraid of VTE recurrence or bleeding? - Which do you fear more? - Did having/Would having a bleed during anticoagulant treatment effect your fears? - What does it feel like to be at risk of recurrent VTE? - What does it feel like to be at risk of bleeding? - Did you know someone who was on an anticoagulant before you started it? If so, did it influence your decision to continue/stop the medication, and how? |

*Individual considerations regarding the decision to stop or continue anticoagulant treatment*

| Personal preference | - What is your own preference regarding treatment duration, and why? - What were the major factors influencing your decision? |
| --- | --- |

*Baseline*

| Demographics | - Age - Sex - Ethnicity - Level of education - Current occupation - Marital status / children |
| --- | --- |

*Are there any aspects that are important to you in this context which have not been discussed?*

**Supplemental Table. Patient Perspectives on Decision-Making for Anticoagulant Duration in Unprovoked VTE**

| **Themes** | **Quotes** |
| --- | --- |
| Impact of bleeding | “But she said, well, I'm going to give you some information now [during a cognitive assessment after subdural hemorrhage]. And later on in the test I'm going to come back to the information I have given you and ask you to repeat it. Well, she went on with the test and then she said OK. Now I'm going back to what I told you at the beginning. Could you tell me what I told you? No. It just was not there. Like it was poof.” |
| Initial experiences when diagnosed with VTE | "I didn't know…until if they hadn't done the CT scans, I would have never known that I even had blood clots. That wasn't something that was ever on the table for me to ever think about." |
| No shared decision-making or explanation | “You know thrombosis is focused on one thing is, you know, stop that clot, right? Keep that blood flowing, right? And I was never provided that kind of option [of stopping anticoagulation], but in terms of decision, warfarin versus Xarelto. I had enough information and, I believe we had that discussion."  “At no point in time did they say we will keep you on it for this amount of time and then take you off it and monitor you closely. They don't seem to have that.”  "And so I said, I've got this balance here and if it means taking this pill longer, I'm OK with that. What didn't come out or what I may not have heard is that there are perhaps [bleeding] risks that go up with continuing this pill for any length of time beyond that three to six month period." |
| Explanation with directive advice | "[The physician] basically explained, Yeah, there's more of a risk because of the UC flare up of ongoing potential to get more clots."  “"But in my recollection. There was no, uhm, options and I can remember talking to him well. You know, they said you'll need to be on blood thinners and I said, well, for how long and their answer was, well, you can't really can't go off it. Because you'll get a clot, you know” |
| Adequate explanation to inform shared decision-making | "Yeah, I thought [the physician] gave me a good amount of information. I guess yeah, I did kind of feel like it was kind of a no brainer." |
| Comfortable going against advice | "Now I did always try and push them to reduce the amount. And up till just recently with Doctor [name redacted], it’s the first time I've seen him and up until him they wouldn't reduce it. They didn't. They felt you can't. I said, can I break the pills in half and try half and then the doctors weren't buying that, but I convinced Dr. [name redacted]. He let me drop it to half the dose I'm taking, right? So, I'm always trying to, you know deal with the risk of another clot, who knows, or even if I will get one. But we're, you know, trying to get the blood thinner down to as low as possible. |
| Follows advice | "I guess I just felt comfortable enough to have faith in the recommendation of the specialist. The person is this specialist, I assume they're gonna take my health and my best interest [into account as much] as possible and give me the solutions that are available for me. But I didn't feel that not taking anything was a viable solution for me at that time. |
| Not concerned about choice | "No, I wasn't presented with an option to say, “OK, this is the alternative solution if you don't stay on blood thinners.” It was recommended that I continue. And as I said, with the positive reaction that I had to the medication, I felt more comfortable with that recommendation." |
| Suggestions for shared decision-making | "I would want to hear what my clinicians are telling me and warning me about the risks and be able to make a good, informed decision about my body and which risks I'm most concerned about, but I cannot do that if I'm not equipped with a the best possible information and an honest assessment of what those risks are. And I I'm clearly not qualified to determine essentially what those risks are.”  “When you're diagnosed with something that's potentially life threatening no matter what it is, you're in a very vulnerable state. You're in a fragile state, emotionally and mentally, and trying to figure out what it all means. And the advantage to where I'm at now is that fragility and that vulnerability isn't there. I feel like my normal self. I'm doing my normal activities. My life is almost like it was other than taking the pill and being conscious and being careful of not chopping a finger off or something like that. I have my life back, so that does afford you a different perspective. So I’m interested in this from a cognitive standpoint in order to make a well informed decision, but I don't feel that vulnerability and that fear in that fragility that I had in the moment, which is a very kind of lonely place to be.” |
| Impact of virtual care during COVID-19 pandemic | "I wish they had [given] me more [information]. That's the hard part when we are doing [consults] over the phone. Doctor calls you and you don’t always get the opportunity to ask what you need to ask. Because they're on a schedule, which I understand and everything, but if you’re in the office, you might get the chance to just say “oh, by the way, I was thinking” and then get a chance to speak. So yeah, it's been different being doctored over the phone rather than being doctored in person.” |
